# Staged management of giant traumatic abdominal wall defect: A rare case report

**DOI:** 10.4103/2321-3868.123077

**Published:** 2013-12-18

**Authors:** Somendra Bansal, Sanchit Jain, Laxmi Narayan Meena

**Affiliations:** 1 Department of General Surgery, Sawai Man Singh Medical College and Hospital, Jaipur, Rajasthan India; 2 6 Kailash Vihar, Lal Kothi, Tonk Road, Jaipur, 302 015 Rajasthan India

**Keywords:** Abdominal wall disruption, blunt trauma abdomen, evisceration

## Abstract

Blunt traumatic abdominal wall disruptions associated with evisceration (abdominal wall injury grade type VI) are very rare. We describe a case of large traumatic abdominal wall disruption with bowel evisceration and complete transection of jejunum and sigmoid colon that occurred after a 30-year-old male sustained run over injury to abdomen. Abdominal exploration and primary end to end jejuno-jejunal and colo-colic anastomosis were done. Staged management of giant abdominal wall defect was performed without any plastic reconstruction with good clinical outcome.

## Introduction

Traumatic abdominal wall hernia (TAWH) associated with blunt injury mechanism is very rare, with an approximate prevalence of 0.2%–1% in majority of reported series.[1,2] Abdominal evisceration (AE) associated with TAWH is even less common with one study reporting an incidence of approximately 1 in 40,000 trauma admissions.[3] Various procedures exist to reconstruct this defect with varying results, availability, and cost. In this report, we describe a case of large traumatic abdominal wall disruption with bowel evisceration following which staged management of giant abdominal wall defect was done.

**Table:** 

Access this article online	
**Quick Response Code:**	**Website:** www.burnstrauma.com
	**DOI:** 10.4103/2321-3868.123077

## Case report

A 30-year-old male presented to emergency department after being involved in run over injury by truck. Examination revealed complete avulsion of skin and subcutaneous tissue from bilateral inguinal region up to bilateral nipples. Upon reflection of avulsed skin, a full thickness defect of size 25 × 20 cm with complete loss of sheath, muscle, and peritoneum was noted in anterior abdominal wall. Through the defect, intestines were protruding out. Tyre marks were present over whole of the avulsed skin. Other positive finding on the physical examination included wound in lateral aspect of left thigh approximately 10 × 5 cm in size. The bowel was easily reduced and saline-soaked gauze was used for temporary coverage over the wound.

**Figure 1:**
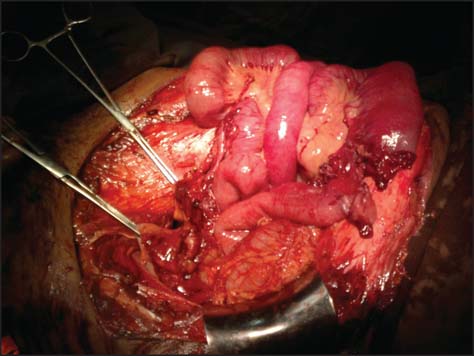
Image showing transected jejunal and colonic loops during the first stage operation.

After resuscitation with intravenous fluids and blood components, patient was taken to the operating room for exploratory laparotomy. Exploration of abdominal cavity was done through the defect. There was complete transection of jejunum about 2.5 feet distal to duodenojejunal flexure and mesentery at that level was incompletely transected with bleeding [Figure 1]. There was also complete transection of sigmoid colon and retroperitoneal hematoma present at right paracolic gutter.

**Figure 2:**
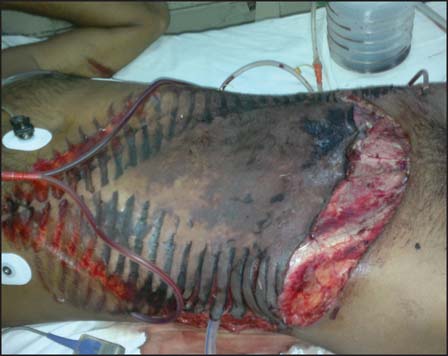
Image showing ingavulsed skin with tyre mark and drains after the first stage operation.

**Figure 3:**
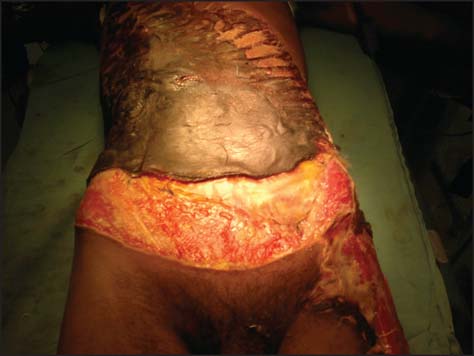
Image showing necrosis of avulsed skin before second stage operation.

After abdominal wash out, patient underwent primary end to end jejuno-jejunal and colo-colic anastomosis in single layer. After abdominal wound debridement, defect was covered temporarily with urobag (urine collecting bag) by taking continuous sutures with margin of defect and urobag [Figure 2]. This was done to prevent abdominal compartment syndrome. After that avulsed skin and subcutaneous tissue was repositioned over the defect. In postoperative period, patient developed necrosis of avulsed skin [Figure 3].

**Figure 4:**
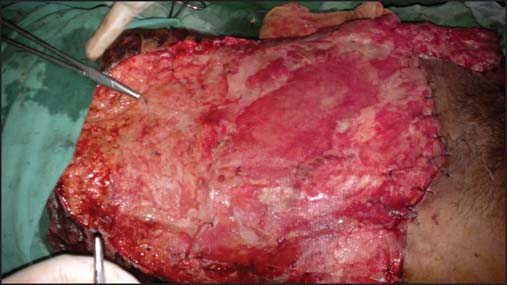
Image showing vicryl meshes being put over granulation tissue after the second stage operation.

On day 12^th^, second stage operation was planned which involved excision of whole necrosed skin and removal of the urobag. We found that whole of the defect was covered with healthy granulation tissue. Two vicryl meshes of 15 × 15 cm size were used to give the strength to the granulation tissue [Figure 4].

**Figure 5:**
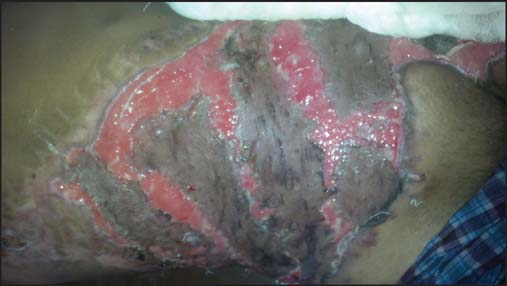
Two weeks after skin grafting (Third stage operation).

Later on vicryl meshes got infected and were removed. Infected wound was managed with saline dressing and antibiotics. Patient’s wound was well covered with healthy granulation tissue with no cough impulse. Therefore, we decided for skin grafting of wound. On day 37^th^, split skin grafting was done over healthy granulation tissue that was taken up very well [Figure 5].

The patient was discharged from the hospital 7 weeks after the initial injury and did well in follow-up at 2 months, both from cosmetic and functional perspective. Further, there was no evidence of hernia at the site of the traumatic defect.

## Discussion

The anterior abdominal wall is a complex fasciomuscular structure that poses a challenge to the reconstructive surgeons. Defects produced from infection, herniation, tumor extirpation, or trauma are among the most often encountered.

The reported incidence of all abdominal wall injuries following blunt trauma in about 9%.[4] TAWHs are thought to result from simultaneous surge in abdominal pressure and the presence of shearing forces that synergistically disrupt the abdominal wall musculature and fascial layers.[5]

AEs constitute an extreme form of TAWHs with the main difference between the two being the amount of force that is focally delivered to the abdominal wall tissues as well as the anatomic location of the force application (i.e., eviscerations tend to occur at anatomically weak points-the lateral rectus, lower abdomen, and inguinal region).[2]

Dennis *et al.*,[4] described an abdominal wall injury scale based on computed tomography (CT) scan finding with overall injury severity graded on scale from I to VI [Table 1]. Of note, among the 140 patients with CT-diagnosed abdominal wall injuries in the study only three had TAWH (grade V injury) and none of the patients had grade VI injury (i.e., complete abdominal wall disruption with evisceration).

**Table 1:** Abdominal wall injury grading scale

Grade	Description
I	Subcutaneous tissue contusion
II	Abdominal wall muscle hematoma
III	Singular abdominal wall muscle disruption
IV	Complete abdominal wall muscle disruption
V	Complete abdominal wall disruption with herniation of abdominal contents.
VI	Complete abdominal wall disruption with evisceration

Our case report describes a case of grade VI injury, an exceedingly rare finding with staged management of giant abdominal wall defect. A case with grade VI injury was also described by McDaniel *et al.*[5]

Regardless of the presence of any associated injuries, prompt surgical repair of the TAWH and/or AEs is required.[2,4] At times, immediate abdominal wall reconstruction may not be possible and staged abdominal wall closure may be required.[4]

Staged management of patients with giant abdominal wall defect without the use of permanent mesh results in a safe and consistent approach for both initial and definitive management with low morbidity and no technique related mortality.[6] Absorbable mesh (like vicryl mesh) provides effective temporary abdominal wall defect coverage with a low fistula rate.[6]

Polypropylene mesh has been used for temporary closure but has been associated with infection, mesh extrusion, and enteric fistula formation. Fistula rates of 12%–50% have been reported when prostheses are used for acute management.[7]

While these infectious complications appear to occur less frequently with the use of absorbable material, these meshes finally lead to an incisional hernia, requiring repair with non-absorbable mesh after a period of 6–12 months. Nevertheless, in the complex situation requiring a temporary abdominal wall closure, use of absorbable mesh material is common.[6,8]

The use of plastic materials (intravenous fluid bags or urine collection bags used as Bogota bag) for closure has been reported by many institutions.[9] The appeal of this material is that it is cheap and easily available in emergent circumstances and therefore it has special significance for low income countries. Generally a sterilized, 3 L genitourinary irrigation bag is sewn to the skin or fascia of the anterior abdominal wall, this is called Bogota bag. It is a temporary abdominal closure technique used to postpone definite closure until predisposing factors causing pathologic elevation of intra abdominal pressure are resolved. It leads to a tension free closure and avoids evisceration and loss of fluids. Also, the abdominal contents can be visually inspected which is particularly useful in cases of ischemic bowel.

Large, full thickness defects require treatment with dressing and nutritional support. From 7 to 20 days from the onset of treatment, exposed viscera become densely adherent to granulation tissue upon which skin graft can be placed. For large abdominal wall defect, which cannot be primarily closed, this technique is safe and has a satisfactory outcome.

In literature, various complications of TAWH include wound infection, abdominal compartment syndrome leading to multiple organ dysfunction, enteric fistula formation. Most important late complication is development of ventral hernia. In our case, only wound infection was seen and during a short-term follow-up of 2 months no hernia was observed.

Definitive closure in such cases is done about 6–12 months later when a planned ventral hernia is operated. Various techniques for repair include component separation method, prosthetic mesh, and flap repair.

## Conclusion

This report describes a case of grade VI injury (i.e., complete abdominal wall disruption with evisceration) which is an exceedingly rare finding along with staged management of giant abdominal wall defect without any plastic reconstruction, without any hernia at the site of injury and with both satisfactory cosmetic and functional outcomes.
